# Effects of *Lacticaseibacillus paracasei* L9 on Oral Microbiota and Cariogenic Factors in *Streptococcus mutans*-Infected Mice

**DOI:** 10.3390/foods13244118

**Published:** 2024-12-19

**Authors:** Xinyao Pu, Bing Fang, Jianmin Wu, Zhi Zhao, Yue Liu, Jingyu Li, Haina Gao, Ran Wang, Ming Zhang

**Affiliations:** 1 School of Food Science and Engineering, Tianjin University of Science and Technology, Tianjin 300457, China; 22842008@mail.tust.edu.cn; 2 Beijing Advanced Innovation Center for Food Nutrition and Human Health, College of Food Science & Nutritional Engineering, China Agricultural University, Beijing 100083, China; bingfang@cau.edu.cn (B.F.); jianminwu@cau.edu.cn (J.W.); zxz0604@yeah.net (Z.Z.); 3 School of Food and Health, Beijing Technology and Business University, No. 11 Fucheng Road, Beijing 100024, China; liuyue2132@163.com (Y.L.); ljy110808@163.com (J.L.); gaohaina103@126.com (H.G.)

**Keywords:** *Lacticaseibacillus paracasei*, dental caries, *Streptococcus mutans*, oral health

## Abstract

In the pathogenesis of dental caries, *Streptococcus mutans* (*S. mutans*) plays a central role. *S. mutans* can produce extracellular polysaccharides, which can help the bacteria form biofilms on the tooth surface, create a stable living environment, and hinder the removal of bacteria by natural defense substances in the oral cavity such as saliva. Meanwhile, the oral microbiota and dietary habits exert long-term influences on its development. This study, employing the BALB/c mouse model, explored the effects of *L. paracasei* L9 on dental caries. In the experiment, mice underwent the *S. mutans* inoculation and were subsequently treated with *L. paracasei* L9 or *S. salivarius* K12 for 28 consecutive days. The results showed that *L. paracasei* L9 significantly ameliorated early enamel caries, and both *L. paracasei* L9 and *S. salivarius* K12 cooperatively downregulated the expressions of critical cariogenic factors, effectively suppressing the initial adhesion of *S. mutans* and the formation of dental plaques. *L. paracasei* L9 reshaped the oral microbiota of caries-affected mice, selectively reducing pathogens abundances and augmenting abundances of probiotics such as Lactobacillaceae and *Streptococcus salivarius*. This study offers a strategic approach for the management of dental caries, highlighting the potential of these probiotics in the field of oral health.

## 1. Introduction

Caries is a microbial-mediated oral disease that causes demineralization and destruction of tooth. The global epidemiological survey of oral health showed that dental caries and periodontal disease are the two most important factors for extraction or removal of tooth. Dental caries is the major cause for loosing tooth in early childhood [1]. It has been listed by the World Health Organization as the third major chronic non-communicable disease after cancer and cardiovascular diseases.

Dental caries is a polymicrobial biofilm disease driven by microbiota–matrix interactions that occur on a tooth surface [2]. Mature dental plaque biofilm is composed of various bacteria embedded encapsulated by matrix that adheres to each other or adheres to the surface of the tooth, between teeth, or the surface of the prosthesis. The major matrix components in oral biofilms associated with dental caries are water-insoluble polysaccharides, particularly *Streptococus mutans* (*S. mutans*) derived glucans. *S. mutans*, an indigenous bacterium in the mouth, is widely regarded as the most important cariogenic microorganism because of its strong capability of initial adhesion, producing glucans, and reducing the pH value. However, *S. mutans* is not the only pathogenic bacteria and other bacterial species might be responsible for biofilm initiation and development.

Recent evidence suggests that more than 700 bacterial species or phylotypes exist in the oral cavity, of which over 50% cannot be successfully cultivated [3]. Recent advances in high throughput genomics now allow for a comprehensive survey of bacterial species present in the oral cavity. The role of *S. mutans* in the initiation of dental caries may not be as dominant as it was previously assumed [4,5]. Some other bacteria may play a more important role. For instance, the presence and level of *Candida albicans* in saliva were reported to be strongly associated with caries pathogenesis, particularly in children, adolescents, and young adults [6,7,8]. In addition, *Campylobacter showae, Parvimonas micra*, and *Leptotrichia hofstadii* may be considered as potential pathogens for dental caries [9]. Therefore, the most promising way to prevent tooth decay is to re-establish the oral microbiota associated with good health [10]. A large number of existing studies have pointed out that regular consumption of probiotic products can significantly reduce the risks of dental caries by inhibiting cariogenic bacteria in the oral cavity and enrich the oral microbiota. The possible mechanisms behind the beneficial effects of probiotics are buffering the saliva pH, producing bacteriocins and enzymes (glucanase, mutanase and urease), and the ability to compete for adhesion and colonization on the tooth surface [11,12].

Current strategies for caries prevention and management mainly encompass the use of topical fluorides, dietary monitoring, and mechanical and chemical plaque control [13]. Although efficacy, the current methods are unlikely to further reduce the incidence of dental caries. Recently, there has been growing attention on the probiotics for dental caries prevention. Probiotics have been confirmed to inhibit the formation of dental biofilm through interaction with oral pathogens. Clinical data also reveal the regular intake of some live probiotics can reduce the caries risk and prevent the caries development in preschool children [14]. Despite some encouraging results, there is widespread skepticism concerning the use of probiotics as anti-caries strategies. The effect of probiotics is strain-dependent and the precise mechanisms by which probiotics exert anti-caries effects remain to be explored.

In our previous study, *L. paracasei* L9 (L9) is a commensal bacterial strain originally isolated from the feces of healthy centenarians, which exhibited a considerable effect on inhibiting *S. mutans* biofilm formation in vitro [15]. However, the effect of *L. paracasei* L9 on dental caries and the action mechanism remain to be explored. In this study, we assessed the inhibitory efficacy of *L. paracasei* L9 on *S. mutans*-induced dental caries in male BALB/c mice and further verified its modification on oral microbiota. In addition, the effect of *L. paracasei* L9 on biofilm formation was also assessed in vitro. The *S. salivarius* K12 (K12, commercialized oral cavity probiotic) was used as a reference strain.

## 2. Materials and Methods

### 2.1. Bacterial Strains and Culture Conditions

The *L. paracasei* L9 strain (CGMCC No. 9800) was isolated from centenarians’ fecal samples and grown in MRS broth at 37 °C under aerobic conditions. *Streptococcus mutans* (*S. mutans*) ATCC 25175 was obtained from the China General Microbiological Culture Collection Center and cultured in BHI medium at 37 °C aerobically. The *S. salivarius* K12 strain (BAA—1024, ATCC, Manassas, VA, USA) was cultured in M17 broth at 37 °C under aerobic conditions. After culture, the *L. paracasei* L9 and *S. salivarius* K12 were centrifuged and resuspended in sucrose solutions for daily oral administration. The *S. mutans* cells were resuspended in a physiological saline solution.

### 2.2. Animals and Experimental Procedure

40 male BALB/c mice aged 3 weeks were purchased from Beijing Vital River Laboratory Animal Technology Co., Ltd. (Beijing, China). All experimental animal care and treatment were approved by the China Agricultural University Institutional Animal Care Committee (No. AW01210202-4-3). After one week of quarantine, mice were divided into four groups (*n* = 10): control group, model group, *L. paracasei* L9 group, and *S. salivarius* K12 group. Except for the control group, mice in other groups were subjected to *S. mutans* incubation, a high sucrose diet (Diet 2000, Trophic Animal Feed High-tech Co., Ltd., Nantong, China), and the 10% (*w*/*v*, g/mL) sucrose drinking water. In order to make the mice develop dental caries, during the model-preparing period, a micropipette was used to inoculate *S. mutans* onto the teeth of the mice for four consecutive days at an inoculation dose of 1 × 10^8^ cells. The mice in the control group were swabbed with sterile PBS. After inoculation, *L. paracasei* L9 or *S. salivarius* K12 was added to drinking water at the concentration of 1 × 10^7^ CFU/mL until the end of experiment. The drinking water was refreshed per 12 h.

### 2.3. Dental Caries Scores

Mice were sacrificed at the end of the study and their heads were stripped manually. The mandibles and maxillae were removed, stained in 0.4% murexide solution for 12 h, and hemisected along the mesiodistal sagittal plane using a diamond-coated band saw with the diameter of 25 mm and thickness of 0.1 mm (Struers Minitom, Copenhagen, Denmark). Smooth surface lesions and sulcal lesions were scored according to a previously published Keyes method [16].

### 2.4. Oral Microbial Counting

A total of five pairs of *S. mutans* primers are shown in Table 1. Using quantitative PCR, we screened for the primers that could specifically amplify the variant streptococci and *Streptococcus oralis*, but not the *Streptococcus salivarius* and *Lactococcus lactis* subsp. *lactis*. PCR amplification of pathogenic bacteria was performed using specific primers, and the products were subjected to agarose gel electrophoresis, gel cutting, and DNA recovery using a gel recovery kit. The recovered PCR products were connected to the pESI-T vector with the Ampicillin resistance gene. The pESI-T vector was transformed into receptive *E. coli* DH5α. After the culture in LB with Ampicillin, culture liquids with gradient concentrations were extracted from the DNA and quantitative PCR was performed using corresponding primers. Meanwhile, the bacterial concentrations of culture liquids were confirmed by gradient dilutions and LB agar medium counting. Then, the standard curves were established between the CT value and bacterial number. Using a similar method, after the DNA extraction from the oral microbial samples and quantitative PCR, the standard curves were used to calculate the abundances of pathogenic bacteria. DNA of HA disks was extracted as previously described [17]. The primers for *S. mutans* are listed in Table 2.

### 2.5. RT-qPCR for Gene Expressions of Dental Biofilms

Four days before the sacrifice, plaque-biofilm samples were collected. Sterile oral swabs were used to rub back and forth for 1 min on the teeth crown, the gap of the teeth, and the gums of mice. The RNA extraction and purification were conducted according to the protocol as described previously [24]. Then, the RNA was reversed into cDNA with the commercial kits (MF166-01, Mei5bio, Beijing, China) and RT-qPCR procedure was finished with the commercial kits (MF797-01, Mei5bio, Beijing, China) via the QuantStudio5 system (ABI, Wakefield, RI, USA). The primers for *spaP*, *luxs*, *ciaH*, *gtfB*, *gtfC*, *gtfD*, *ldh*, and *recA* are listed in Table 2.

### 2.6. S rRNA Gene Sequencing of Dental Plaques

Plaque microbiota samples were collected one day before the sacrifice. Sterile oral swabs were used to rub back and forth for 1 min in the crown, the gap of the teeth, and the gums of mice. The cotton swabs were then immersed in preservation solutions and stored at 4 °C for DNA extraction. Bacterial genomic DNA was extracted with phenol-chloroform [25], and then quantified by NanoDrop (OneC, Thermo Scientific, Waltham, MA, USA). The V3–V4 region of 16S rRNA gene was amplified with the universal primers as described previously [26]. The products were pooled based on a uniform standard and sequenced on an Illumina Miseq PE300 platform (Illumina, San Diego, CA, USA) by a paired-end sequencing strategy. Raw data were assembled filtered, and then used to select the operational taxonomic units (OTUs) with USEARCH software (version 7.0, http://www.drive5.com/usearch/, accessed on 1 October 2024) based on the 97% sequence similarity. The OTUs were further subjected to the Ribosomal Database Project classifier software (version number: RDP Release 11.5) for taxonomic identification. Further analysis, such as abundances of special communities, was analyzed on the platform of Majorbio Cloud Platform (https://cloud.majorbio.com, accessed on 1 October 2024).

### 2.7. Biofilm Formation Assay

The concentrations of *L. paracasei* L9 cultured in MRS broth medium, *S. salivarius* K12 in M17 broth medium, as well as *S. mutans* in BHI medium with 1% sucrose, were adjusted to OD600 = 0.7 ± 0.05 (5 × 10^8^ cfu/mL) and OD600 = 0.5 ± 0.05 (1 × 10^8^ cfu/mL), respectively. The 24-well cell culture plates were then prepared by adding 1 mL of artificial saliva medium containing 1% sucrose, 50 μL of *S. mutans* suspension, and 50 μL of probiotics suspension. The control group consisted of equivalent MRS or M17 broth medium. The plates were incubated under anaerobic conditions at 37 °C for 24 h. Subsequently, the liquid culture medium was carefully aspirated with a pipette, and the cells were washed 3 times with PBS. The floating *S. mutans* cells without forming films were removed. The plates were then air-dried. For each well, 100 μL of 99% methanol was added and treated the cells for 15 min. Then, the supernatant was aspirated, and the plates were air-dried. Next, 200 μL of 0.1% CV solution was added to wells for 20 min, followed by 3 washes with PBS to remove redundant CV. Finally, 1 mL of 33% acetic acid was added to release the bound CV, and the absorbance was measured at 595 nm using an ELISA reader (Perkin Elmer Victor X3, Shelton, CT, USA).

### 2.8. SEM Observation of Biofilms

Microbial biofilms were grown on 14 mm diameter round glass slides (SORFA, China) in the wells of 24-well plates. After 24 h of anaerobic culture, the liquid culture medium was gently aspirated, and the glass slides were removed. To wash off the floating *S. mutans*, PBS was used to wash. The *biofilms* were then fixed in 2.5% glutaraldehyde at 4 °C overnight. The glass slides were successively dehydrated with 30%, 50%, 70%, 80%, and 90% ethyl alcohol for 1 h. Subsequently, the dried glass slides were stuck onto the sample table, sprayed with gold, and observed using SEM (HITACHI, S-4800, Tokyo, Japan).

### 2.9. Statistical Analysis

Data are expressed as the mean ± SEM. Statistical comparisons were made using one-way analysis of variance (ANOVA) with Dunnett’s multiple comparisons. A *p* value of less than 0.05 is considered significant. Statistical analysis was performed with SPSS 18.0 (SPSS, Inc., Chicago, IL, USA). Graphs were generated by GraphPad Prism 7.0 (GraphPad Software, San Diego, CA, USA).

## 3. Results

### 3.1. Dental Caries Scores

**Figure 1:**
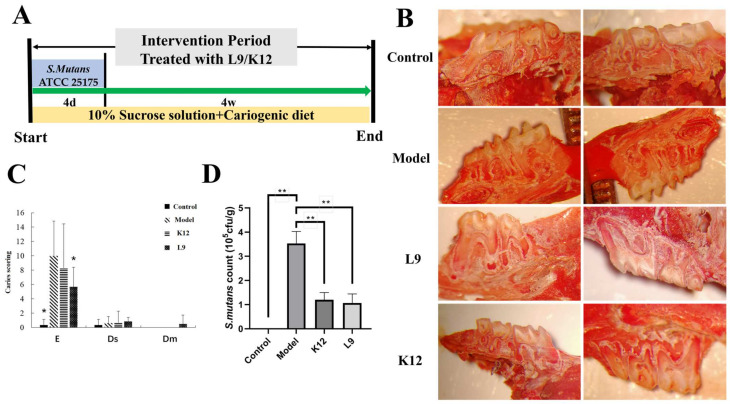
Effects of *L. paracasei* L9 and *S. salivarius* K12 on dental caries in mice. (**A**) The process and modeling methods of this experiment. (**B**) Schematic diagrams of the occlusal surface of mouse teeth. (**C**) Grade E, Ds, and Dm grade caries scores. (**D**) The *S. mutans* count in oral cavity. * *p* < 0.05, ** *p* < 0.01, versus model group.

The process and modeling method of this experiment are shown in Figure 1A. Figure 1B illustrates the main effects of *L. paracasei* L9 and *S. salivarius* K12 treatments on the occlusal surface of mice teeth. As shown, the occlusal surface of the control group’s mice teeth is smooth, lacking deep grooves, whereas the model group’s occlusal surface displays evident caries, with almost every tooth having deep sulci on its occlusal surface (Figure 1B). Probiotic strains *L. paracasei* L9 and *S. salivarius* K12 considerably obstructed the cariogenic actions of *S. mutans*, leaving the teeth’s occlusal surface relatively intact, yet small numbers of superficial caries remained present (Figure 1B). On the occlusal surfaces of the mice’s teeth, E-level, Ds-level, and Dm-level caries were detected (Figure 1C). In E-level caries (Figure 1C), the model group had a significantly higher caries score than control group (*p* < 0.05), whereas the *L. paracasei* L9 group showed a notably reduced score compared to model group (*p* < 0.05). The *S. salivarius* K12 group had a slightly decreased score than model group, but it was not statistically different (*p* > 0.05). Regarding the Ds and Dm level caries scores for each group, they were all under 1, and hardly any shallow caries could be found, with no notable differences (Figure 1C, *p* > 0.05). Ds and Dm level caries emerged at the outer layers of the enamel and dentin. Pathogenic bacteria, specifically *Streptococcus* abundances in the oral cavity, were also measured in Figure 1D, where the *Streptococcus* count in the model was significantly elevated versus other groups (*p* < 0.05), and both *S. salivarius* K12 and *L. paracasei* L9 groups had drastically lower counts than the model group (*p* < 0.05). Overall, these results suggest that *L. paracasei* L9 has a marked preventative effect on initial stage caries on the teeth’s occlusal surface.

### 3.2. Quantification of Caries-Causing Factors

**Figure 2:**
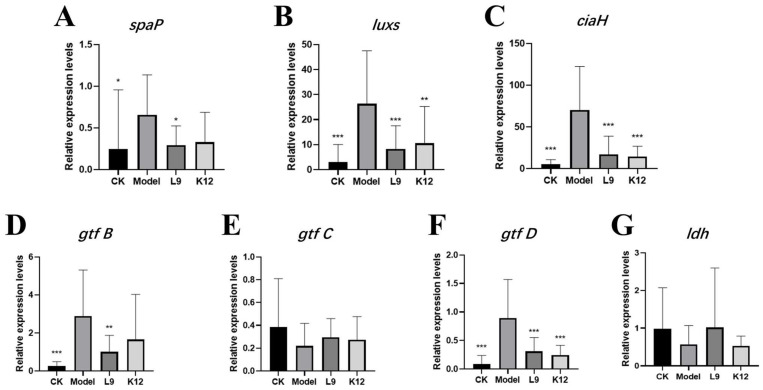
The gene expressions of caries-causing factors in mouse dental plaque. (**A**–**C**) The relative expression histograms of *spap* (**A**), *luxs* (**B**), and *ciaH* (**C**) genes. (**D**–**F**) The relative expression histogram of *gtf* gene. (**G**) The relative expression histogram of *ldh* genes. * *p* < 0.05, ** *p* < 0.01, *** *p* < 0.001, versus the model group.

The quantitative results of caries-causing factors in mouse dental plaque are shown in Figure 2. It can be seen that compared to the control (CK) group, the relative expressions of *spap*, *luxs*, and *ciaH* in the model group were significantly higher (Figure 2A–C, *p* < 0.05), the expression levels of *spap* and *ciaH* in the *L. paracasei* L9 and *S. salivarius* K12 groups were reduced compared to the expression levels of the CK group, and the expression of *Luxs* gene was significantly lower than that of the model group (Figure 2B, *p* < 0.05). But compared with the CK group, the mean relative expression levels were still increased, especially in the *L. paracasei* L9 group (Figure 2B).

As shown in Figure 2D–F, the relative expressions of *gtfB* and *gtfD* were significantly higher in the model group compared to the CK group (*p* < 0.05), especially the *gtfB*. The expressions of both genes in the *L. paracasei* L9 and *S. salivarius* K12 groups were significantly lower than those in the model group (Figure 2D–F, *p* < 0.05) except the *gtfB* expression in the *S. salivarius* K12 group, although the mean expression levels of both genes were increased compared to the CK group. In addition, the relative expressions of *gtfC* were not significantly different among the four groups (Figure 2E, *p* > 0.05).

The *ldh* gene encodes lactate dehydrogenase. As can be seen from the results in Figure 2G, the mean relative expression level of *ldh* was relatively low in the *L. paracasei* L9 group, and the relative expression level of *ldh* was not significantly different among the four groups (*p* > 0.05). Therefore, *L. paracasei* L9 may show no significant effect on the acid product capacity of *S. mutans*.

### 3.3. Effects of L9 and K12 on Oral Microbial Community

For the α-diversity of oral microbiota, evaluated by the Sobs, Shannon, Simpson, Ace, and Chao indexes, there was no significant difference in each index between the model and control groups (Table 3, *p* > 0.05), meaning both diversity and richness did not show a significant change. In the *S. salivarius* K12 group, the Simpson index was obviously lower than that of the model group, and the Chao index was increased (Table 3, *p* < 0.05), indicating the increased α-diversity. There was no significant difference in each index between the *L. paracasei* L9 and the model groups (Table 3, *p* > 0.05), suggesting the not significant impact on oral bacteria diversity and richness.

**Figure 3:**
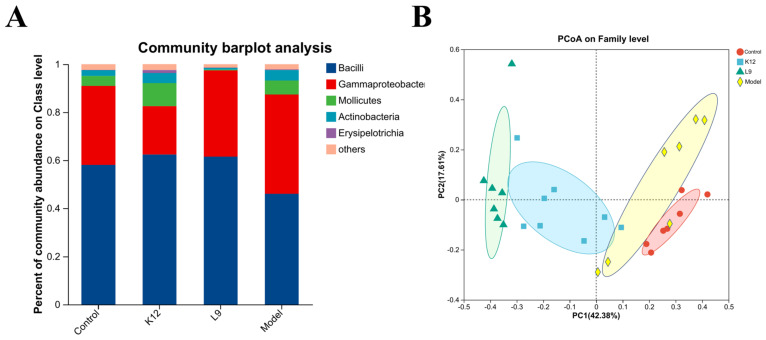
Effects of *L. paracasei* L9 and *S. salivarius* K12 on oral microbial structure. (**A**) Percent of community abundance on Class level. (**B**) PCoA on Family level.

To detect the compositions of the dental plaque microbiota in mice, the third generation PacBio sequencing technology based on the full-length bacterial 16S rRNA gene was adopted, which can identify bacteria at the species level. At the class level, the top five classes with the highest abundances were presented, including Bacilli, Gammaproteobacteria, Mollicutes, Actinobacteria, and Erysipelotrichia (Figure 3A). Principal Coordinate Analysis (PCoA) based on Bray–Curtis distance is a method used to evaluate the similarities in the compositions of microbiota communities (Figure 3B). According to the results, the model group was almost completely separated from the other three groups. Meanwhile, there was no overlap between the control group and the *L. paracasei* L9 group, as well as the *S. salivarius* K12 group (Figure 3B).

**Figure 4:**
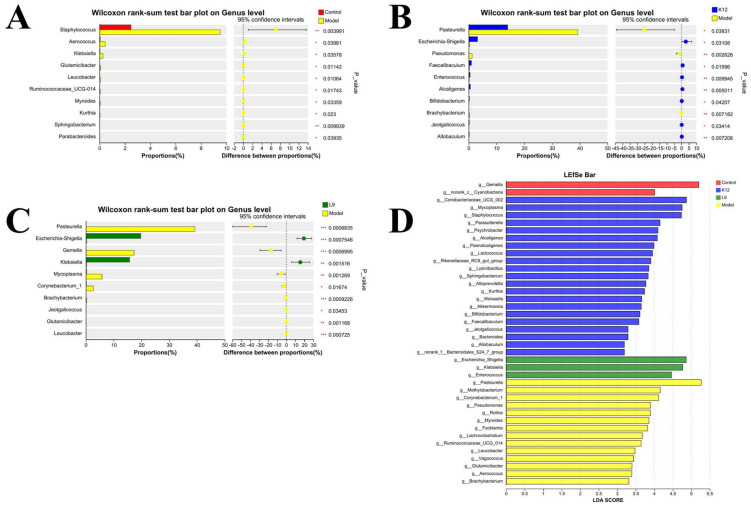
Analysis of bacterial abundance differences between the *L. paracasei* L9 or *S. salivarius* K12 or control group and model group. (**A**) Bacterial abundance differences between the control and model groups. (**B**) Bacterial abundance differences between the *S. salivarius* K12 and model groups. (**C**) Bacterial abundance differences between the *L. paracasei* L9 and model groups. (**D**) The LEfse results of *L. paracasei* L9, *S. salivarius* K12, control, and model groups. * *p* < 0.05, ** *p* < 0.01, *** *p* < 0.001.

The control, *S. salivarius* K12, and *L. paracasei* L9 groups were compared with the model group in order to analyze different bacteria at the genus level. Compared with control group, the model group showed higher abundances of *Staphylococcus*, *Aerococcus*, and *Klebsiella* (*p* < 0.05). Similarly to the *S. salivarius* K12 group, compared with the model group, the *L. paracasei* L9 group had a significant decrease in the abundance of *Pasteurella* and a significant increase in the abundance of *Escherichia—Shigella* (Figure 4B,C, *p* < 0.05). The difference is that, compared with the model group, the *L. paracasei* L9 group showed a significant decrease in the abundances of *Gemella, Mycoplasma*, and *Corynebacterium_1*, while the *Klebsiella* increased significantly (Figure 4B,C, *p* < 0.05). The Linear Discriminant Analysis Effect Size (LEfse) was used to evaluate the influences of significantly different species among different groups. As we can see, *Gemella*, *Coriobacteriaceae_UCG_002*, *Escherichia_Shigella*, and *Pasteurella* were the communities with the highest LDA scores in the control, *S. salivarius* K12, *L. paracasei* L9, and model groups, respectively (Figure 4D).

### 3.4. Biofilm Staining

**Figure 5:**
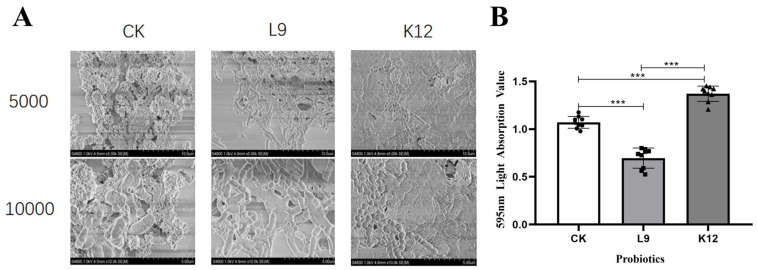
Effects of *L. paracasei* L9 and *S. salivarius* K12 on formation of *S. mutans* biofilms. (**A**) SEM images of *S. mutans* biofilms after a 24 h treatment with probiotics. 5000 and 10,000 are magnification. (**B**) The absorbance values after the staining of *S. mutans* biofilm. *** *p* < 0.001.

Figure 5A shows the SEM images of *S. mutans* biofilms after the 24 h of treatment with probiotics, we viewed its microstructure using scanning electron microscopy (SEM) under the 10 μm or 5 μm scales. The top and bottom are the *S. mutans* biofilms of the control (CK), *L. paracasei* L9, and *S. salivarius* K12 groups. It can be seen that the bacteria of the CK group are enveloped by the biofilms of *S. mutans*, while the multilayer dense biofilm structure of the *L. paracasei* L9 group disappears (Figure 5A). Instead, the biofilms show a single layer of loose microstructure (Figure 5A). The rod-like structure of *L. paracasei* L9 is completely exposed to the biofilms, presenting the dense and discontinuous biofilms with a certain thickness (Figure 5A). In *S. salivarius* K12 group, the *S. salivarius* K12 is mixed with *S. mutans* to form the dense biofilms (Figure 5A).

Figure 5B shows the absorbance values of biofilm staining of *S. mutans* after the 24 h of treatment with probiotics. It is obvious that the absorbance value of the *L. paracasei* L9 group was lower and that of the *S. salivarius* K12 group was higher compared to the CK group. *L. paracasei* L9 inhibited the promotion of biofilm formation, while *S. salivarius* K12 promoted the participation of *S. mutans* in the biofilm formation.

## 4. Discussion

The probiotics can prevent or mitigate dental caries, which is a well-established concept in the field of oral health [27]. Notably, *Lactobacillus plantarum* has been shown to effectively treat caries caused by *Candida albicans* [28], highlighting its therapeutic potential. Additionally, *Lactobacillus rhamnosus* has been found to continuously regulate the cariogenic potential of *S. mutans* by inhibiting acid production, thereby mitigating the risk of dental caries [29]. In recent years, the role of *Lacticaseibacillus paracasei* in alleviating dental caries has also been progressively elucidated, including its ability to suppress the formation of *S. mutans* biofilms and production of bioactive substances, as well as enhancing the levels of salivary neutrophil peptides [30,31]. This study contributes to the expanding corpus of knowledge by demonstrating the efficacy of the *L. paracasei* L9 strain in mitigating the cariogenic phenotype in a murine model. The mitigation manifests as a diminishment in caries damages, reduced expressions of cariogenic factors, and inhibition of initial *S. mutans* adhesion [32]. While other strains of *Lacticaseibacillus paracasei* have been identified to possess anti-caries effects [33], this investigation singularly identified *L. paracasei* L9 as uniquely capable of alleviating caries damages and inhibiting initial *S. mutans* adhesion. This finding provides a more robust strategy for incorporating *L. paracasei* L9 into functional foods, particularly those targeted at oral health products, thereby enhancing its application within the realm of oral healthcare [34].

Biofilms, a collective lifestyle of microorganisms, exhibit a myriad of emergent properties encompassing interfacial aggregation, surface adhesion, water retention, nutrient acquisition, enhanced resistance, and collective coordination [35,36,37,38,39]. Upon formation, bacterial biofilms serve as a sanctuary for microorganisms, significantly amplifying their resilience against antimicrobial agents and their ability to evade host’s immune system, thereby facilitating recalcitrant and recurrent infections. Within the oral cavity, dental caries arises from biofilms formed by the pathogenic *S. mutans* on the initial proteinaceous coating of the dental matrix [40,41,42]. Consequently, the inhibition of bacterial biofilm formation, particularly that of *S. mutans*, is vital for the preservation of oral health. In light of the adverse effects of antimicrobial agents, biocontrol strategies have emerged as a promising alternative. This study aimed at elucidating the potential of *L. paracasei* L9 to mitigate dental caries in the oral environment. Utilizing a co-culture system with *S. mutans*, this investigation focused on assessing the impact of *L. paracasei* L9 on the formation of *S. mutans* biofilms. To emulate the oral ecosystem, *S. mutans* were cultivated in artificial saliva medium supplemented with 1% sucrose throughout the experiment [43]. Following a 24 h co-culture period, the viable cells and metabolites secreted by *L. paracasei* L9 exhibited a pronounced inhibitory effect on the assembly of *S. mutans* biofilms. This finding indicates that live *L. paracasei* L9 can effectively suppress the development of *S. mutans* biofilms through the secretion of its metabolites, thereby offering a protective barrier against dental caries. The gene *Spap* is regulated by a surface protein P1 secreted by caries-causing bacteria that binds specifically to receptors on saliva-acquired membranes, thereby promoting initial bacterial adhesion, the first stage of dental plaque biofilm formation. It has been shown that the relative expression of the *Spap* gene in *S. mutans* with high adhesion capacity is higher than that in bacteria with weak adhesion capacity during initial adhesion, and its significant down-regulation indicate that the addition of *L. paracasei* L9 may inhibit the initial adhesion of *S. mutans* by regulating adhesin-receptor binding. The two-component luxs/AI-2 system in the density-sensing system of *S. mutans* is activated with its increase in *S. mutans* [44]. Luxs is a key enzyme for autoinducer-2 (AI-2), while *luxs* and the *ciaH* gene co-regulation of wooly sulfobacteriocin has been shown to reduce the biofilm forming ability of *S. mutans* with the *luxs* mutation [45]. Similarly to the results of the present study, the downregulation of *Luxs* gene was accompanied by a significant reduction in caries score in mice. Therefore, the addition of *L. paracasei* L9 significantly inhibited the initial adhesion of *S. mutans* to the tooth surface. With the development of biofilms, *S. mutans* convert carbohydrates from oral intake into extracellular polysaccharides via glucosyltransferases, which promotes bacterial adhesion mediated by insoluble polysaccharides. Glucosyltransferases (GTF) are regulated by *gtf* genes, of which *gtfB* and *gtfC* genes are closely associated with sucrose-dependent adhesion in *S. mutans*. It has been shown that the relative gene expressions of *gtfB*, *spaP*, and *luxS* in *S. mutans* were significantly upregulated in the biofilms. Moreover, the expressions of *gtf* genes in biofilms of *S. mutans* were higher than the planktonic state. This result was slightly different from that of Wudi-Cong et al. [46,47]. Their results showed that the expressions of the three *gtf* genes of *S. mutans* were consistently upregulated during caries formation. The decrease in *gtfD* expression may reduce the availability of soluble sugars for GTF-D synthesis and thus reduce the availability of GTF-B metabolic substrate. Therefore, the decreases in relative expressions of *gtfB* and *gtfD* genes suggest that *L. paracasei* L9 significantly inhibits the formation and development of dental plaque biofilms.

Dental caries, a multifactorial chronic disease, is closely associated with the disruption of oral microbial homeostasis [48]. The oral microbiota, composed of billions of microorganisms, is one of the most intricate microbial ecosystems in the human body [49]. Under normal circumstances, these microbes co-exist in a delicate balance that sustains oral health. However, when this equilibrium is disrupted, particularly when the oral environment favors the overgrowth of certain pathogens, such as the *S. mutans*, the onset of oral diseases like caries becomes inevitable [50,51]. In this experiment, the introduction of *L. paracasei* L9 significantly optimized the oral microbial structure, inhibiting the overgrowth of potential pathogens such as *Pseudomonas aeruginosa*, *Veillonella*, *Klebsiella*, and *Gemella*, while simultaneously promoting the proliferations of beneficial bacteria from the *Lactobacillus* genus and *Streptococcus salivarius*. This positive ecological regulation effectively reduced the risks of dental caries, periodontal disease, halitosis, and oral tumors, and also had a positive impact on the prevention of systemic diseases such as sepsis, highlighting the significant potential of *L. paracasei* L9 in the maintenance of both oral and systemic health [52,53,54]. Through the intervention of *L. paracasei* L9, the oral microbial environment is optimized, the balance of microbiota is restored, thus offering a safe, natural, and effective approach for oral health maintenance. This study not only underscores the potential of probiotics in the prevention and treatment of oral diseases, but also provides a new perspective on exploring the links between oral diseases and overall health.

In the model group, the abundances of pathogenic bacteria in the *L. paracasei* L9 group decreased significantly. For example, the abundances of *Pasteurella* and *Gemella* decreased. In contrast, the abundance of beneficial *Klebsiella* increased significantly. Relatively, the most bacteria with high abundances in the model group were pathogenic bacteria, such as *Pasteurella* and *Methylobacterium*. However, the abundances of these bacteria in *L. paracasei* L9 group were very low. Therefore, it can be concluded that *L. paracasei* L9 may optimize the oral microbial environment.

This experiment exhibits certain reference significance for the prevention and alleviation of dental caries. However, it still has some limitations. For example, we have not carried out relevant population experiments and have not explored whether *L. paracasei* L9 can inhibite the *S. mutans* biofilms formation through interference quorum sensing. In the future, we plan to recruit a group of toddler volunteers to carry out relevant experiments to observe the influence of *L. paracasei* L9 on their oral microbiota.

## 5. Conclusions

In conclusion, an intervention with *L. paracasei* L9 can repress the expressions of cariogenic factors and inhibit the initial adhesion of *S. mutans* and the subsequent formation and development of dental biofilm, mitigating the severity of caries formation and induced damages. Restraining the dysbiosis of oral microbiota is a key functional mechanism. These findings build a solid scientific foundation for the potential application of *L. paracasei* L9 in the prevention and treatment of dental caries.

## Figures and Tables

**Table 1:** The primers screened for specifically recognizing variant *streptococci* and *Streptococcus oralis*.

Number	Forward Primers (5′-3′)	Reverse Primers (5′-3′)	Product Length (bp)
B1	AGCGTTGTCCGGATTTATTGG	AGCGTTGTCCGGATTTATTGG	95
B2	GCCTACAGCTCAGAGATGCTATTCT	GCCATACACCACTCATGAATTGA	180
B3	AGCAATGCAGCCATCTACAAAT	ACGAACTTTGCCGTTATTGTCA	98
B4	ACTGTTCCCCTTTTGGCTGTC	AACTTGCTTTGATGACTGTGGC	93
B5	TGTACCCCGTATCGTTCCTGTG	AAAGACTGGAGTTGCAATGTGAATA	175

**Table 2:** The primers for *caries-causing factors*.

Primers	Forward Sequences (5′-3′)	Reverse Sequences (5′-3′)	Reference
*spaP*	TGATGTTGCTTCTTCTATGGAG	CAGGTTAGTGTATGTAAGCTGT	[18]
*luxs*	CCCTATGTTCGCTTGATTGGGG	AGTCAATCATGCCGTCAATGCG	[19]
*ciaH*	GCGAAGTGGAGTCAAAAACC	AACTCTCCAAGCGATTTTGC	[20]
*gtfB*	CGAACAGCTTCTAATGGTGAAAAGCTT	TTGGCTGCATTGCTATCATCA	[21]
*gtfC*	TGATTAACATGGATAACAGG	CCAAACTGTTAGTGATCAGA	[22]
*gtfD*	CCAATATTCCGACAGCCTATG	TCACCATAATAAAGACGTGTAATTGAA	[23]
*ldh*	CTTGATACTGCTCGTTTCCGTC	GAGTCACCATGTTCACCCAT	[18]
*recA*	GCCTATGCTGCTGCTCTTG	TCACCAATATCTCCGTCAATCTC	[18]
*S. mutans*	GCCTACAGCTCAGAGATGCTATTCT	GCCATACACCACTCATGAATTGA	This study

**Table 3:** Alpha-diversity index difference test table.

Type of Index	Control	Model	L9	K12	*p* Value (Model–Control)	*p* Value (L9–Model)	*p* Value (K12–Model)
Sobs	119.9 ± 62.915	102.57 ± 27.969	84.3 ± 36.314	128.33 ± 28.465	0.508	0.282	0.073
Shannon	1.792 ± 0.261	1.662 ± 0.464	1.659 ± 0.277	1.966 ± 0.284	0.472	0.984	0.093
Simpson	0.259 ± 0.052	0.344 ± 0.133	0.298 ± 0.111	0.241 ± 0.065	0.084	0.450	0.036 *
Ace	130.94 ± 65.275	125.28 ± 31.646	118.6 ± 39.195	161.83 ± 45.61	0.835	0.715	0.080
Chao	132.71 ± 67.921	119.56 ± 29.068	106.95 ± 36.224	153.25 ± 32.305	0.639	0.458	0.036 *

*: *p* < 0.05.

## Data Availability

The original contributions presented in this study are included in the article. Further inquiries can be directed to the corresponding author.
